# Clinical-pathological findings of otitis media and media-interna in calves and (clinical) evaluation of a standardized therapeutic protocol

**DOI:** 10.1186/s12917-015-0606-3

**Published:** 2015-12-03

**Authors:** I. Bertone, C. Bellino, G. L. Alborali, A. Cagnasso, G. Cagnotti, E. Dappiano, M. Lizzi, M. Miciletta, A. Ramacciotti, P. Gianella, A. D’Angelo

**Affiliations:** Department of Veterinary Science, University of Turin, Grugliasco, Italy; Diagnostic Section of Brescia, Istituto Zooprofilattico Sperimentale della Lombardia e dell’Emilia-Romagna, Brescia, Italy; Private Practitioner Turin, Turin, Italy; Private Practitioner Udine, Udine, Italy; Zoetis Italia, Rome, Italy; Private Practitioner Asti, Asti, Italy

**Keywords:** Bovine, Calves, Neurology, Otitis, Therapy

## Abstract

**Background:**

The aims of this field trial were to describe the clinical-pathologic findings in calves with otitis media (OM) and media-interna (OMI), to evaluate, through the development of a scoring system, the effectiveness of a standardized therapeutic protocol, and to identify the causative pathogens and their possible correlation with concurrent respiratory disease. All animals underwent physical and neurological examinations at three experimental time points: at diagnosis/beginning of treatment (T0), 1 week (T1) and 2 weeks (T2) after therapy was started, respectively. Follow-up telephone interviews with animal owners were conducted 1 month later. The therapeutic protocol consisted of tulathromycin (Draxxin®; Zoetis), oxytetracycline hydrochloride (Terramicina 100®; Zoetis), and carprofen (Rimadyl®; Zoetis).

**Results:**

Twenty-two calves were enrolled. Physical and otoscopic examination at T0 revealed monolateral and bilateral otorrhea in 16 and 6 calves, respectively, with peripheral vestibular system involvement in calves presenting with neurological signs (*n* = 17; 77 %). A significant improvement of clinical and neurological scores was observed in 20 (90 %) calves, a full recovery in only 1 (5 %). One calf worsened between T0 and T1 and it was removed from the study. None of the other animals showed a worsening of clinical conditions and/or required further treatments at one month follow up. *Mycoplasma bovis* was isolated in 89 % of the affected ears either alone or together with *P. multocida* (*n* = 5), *Streptococcus* spp. (*n* = 1), *Staphylococcus* spp. (*n* = 1), and *Pseudomonas* spp. (*n* = 1). *M. bovis* either alone or together with these bacteria was also isolated from the upper and/or lower respiratory tract in 19 (86 %) calves.

**Conclusions:**

This is the first prospective study to evaluate the effectiveness of a standardized therapeutic protocol for the treatment of OM/OMI in calves. The therapy led to clinical improvement in the majority of the calves. Persistence of mild clinical-neurological signs did not compromise productive performance. The numerical scoring system for clinical and neurological signs permitted objective evaluation of response to therapy. *M. bovis* was the pathogen most often isolated. This finding should be considered in the treatment of OM/OMI in calves. Moreover, respiratory tract infection should not be underrated, since it is one of the major risk factors for the development of OM/OMI.

## Background

Otitis media (OM) and media-interna (OMI) are common diseases in cattle. They can occur either sporadically or as an outbreak [1], causing economic losses [2]. Young animals, from 1 week to 18 months of age, are usually affected [2, 3], although OM has also been reported in an adult cow [4]. No gender predisposition has been demonstrated but inclement weather has been associated with increased incidence [5–9]. Reportedly, beef calves are more often affected, though the incidence of OMI seems to be rising among dairy calves, as well [6–8, 10, 11]. OMI occurs frequently in association with respiratory infections, and the same causative pathogens have been isolated [1, 8]. The most common route of infection is through the Eustachian (auditory) tube. The pathogens can also reach the middle ear by hematogenous spread or by migration from the external ear, which occurs primarily in cases of parasitic otitis [1, 6, 8, 12, 13]. *Mycoplasma bovis*, either alone or in association with other bacteria, is considered one of the main etiological agents of OMI in calves [1, 8, 10, 13]. Clinical signs of OM and OMI vary from conjunctival discharge, pain, obtundation, poor appetite and pyrexia [5, 7, 8, 14, 15] to neurologic manifestations secondary to facial nerve (cranial nerve VII) and vestibulocochlear nerve (cranial nerve VIII) dysfunctions [1]. Purulent aural discharge may appear into the external acoustic meatus in association with rupture of the tympanic membrane. Increased pressure into the tympanic bulla due to the exudate filling the cavity can cause the membrane to rupture [2, 9]. Complications of OMI are ill thrift and intracranial extension with worsening of neurological signs [8, 11]. To prevent economic losses due to treatment costs and weight faltering, diagnosis should be made during the early or subclinical stages of the disease [12, 16]. The presumptive clinical diagnosis is based on physical and neurological evaluation and can be confirmed by otoscopic examination and other diagnostic imaging techniques [8, 11, 16, 17]. A definitive diagnosis of OM and OMI is reached at necropsy, as well [1]. Treatment in the acute stages has been effective, while poor response to therapy has been related to chronicity or development of complications [1, 2, 8, 12].

Prospective studies on the effectiveness of therapeutic standardized protocols for OM and OMI in calves are lacking to date. The hypothesis of this study were to evaluate if a standardized therapeutic protocol can be used in the treatment of OM and OMI in veal calves and if it is possible to assess objectively the response to therapy. The primary aims of this field trial were (1) to describe the clinical-pathologic findings in calves with OM and OMI and (2) to evaluate, through the development of a scoring system, the effectiveness of a standardized therapeutic protocol. A secondary objective was to identify causative pathogens and their possible correlation with concurrent bovine respiratory disease (BRD) in Italy, in the referred population*.*

## Methods

The study was performed according to ethical recommendations, animal welfare considerations and regulations (Directive 98/58/EC and Italian Decree Law 146/2001). Moreover, for any patient referred at Teaching Hospital of the Department of Veterinary Science of Turin (Italy) the owner or an agent for the owner had to read and sign an informed owner consent in order to authorize any veterinary assessment and treatment needed for his/her animal. Calves referred to the Teaching Hospital of the Department of Veterinary Science of Turin (Italy) between February 2013 and December 2014 for suspicion of acute OM and OMI were selected for the study. Animals were recruited if they had not received treatment, had undergone physical and neurological examination, if OM and/or OMI were confirmed by aural examination by means of otoscopy/endoscopy, and if follow-up records were available. All animals underwent bilateral otoscopic examination with a hand-held otoscope (Heine Beta®TR 3.5 V rechargeable handle, Heine G-002.21.301 slit illumination head, 65 mm × 6 mm diameter closed specula, Heine Optotechnik, Herrsching, Germany); in selected cases, endoscopic examination was carried out using a laparoscope (Karl Storz GmBH, Tuttlingen, Germany, Ottica Hopkins II, 2.7 mm × 15 cm).

### Physical and neurological examination

Physical and neurological examinations were performed at three experimental time points: at diagnosis/beginning of treatment (T0), 1 week (T1) and 2 weeks (T2) after therapy was started, respectively. During follow-up telephone interviews with the owners 1 month after T2, information was collected on the animals’ mental status, appetite, weight gain, possible worsening and further treatments. Physical examination was based on a protocol tested and optimized in a previous pilot study (data not shown). Neurological examination was performed by a board-certified neurologist (ADA). A two-part standardized data collection form, one for physical and one for neurological findings, was completed for each animal. The scoring system for clinical signs was derived from the Calf Respiratory Scoring Chart, University of Wisconsin [18]^1^. The scoring system for neurological signs was devised on the basis of a previous pilot study by the authors. Tables 1 and 2 show the numerical scoring systems used to quantify the clinical and neurological findings, respectively, at each experimental time point. A final total score (clinical + neurological scores) was then obtained for each animal at each time point.

**Table 1:** Classification of severity (score) of clinical findings in calves with otitis

Score	Clinical findings
	Rectal Temperature
0	≤ 39.2 °C
1	39.3 °C < T° < 40 °C
2	≥ 40 °C
	Feces
0	Normal feces
1	Feces softer than normal, but no diarrhea on tail
2	Diarrhea but not profuse, wet tail
3	Profuse watery diarrhea with blood and fibrin, wet tail
	Respiratory System
0	No respiratory signs
1*	Induced or spontaneous cough
1*	Nasal discharge
1*	Eye discharge
*one point for each sign	
	Otoscopic Examination
0	Absence of purulent material
1*	Presence of purulent material
*one point for each side (left/right)	
	Joints
0	No signs of arthritis
1*	Presence of arthritis
*one point for each affected joint

**Table 2:** Classification of severity (score) of neurological findings in calves with otitis

Score	Neurological findings
	Mental Status
0	Normal
1	Depressed
2	Stuporous
	Posture
0	Normal
1	Head tilt
2	Recumbent - unable to stand
	Gait
0	Normal
1	Vestibular ataxia
2	Hemiparesis/Hemiplegia
3	Tetraparesis/Tetraplegia
	Proprioceptive Testing
0	Normal
1	Ipsilateral Decreased proprioceptive positioning reaction
2	Bilateral Decreased proprioceptive positioning reaction
	Cranial Nerve Assessment
0	No cranial nerve deficits
1^a^	Anisocoria
1^a^	Positional strabismus (III, IV, VI, VIII)
1^a^	Patological nystagmus (III, IV, VI, VIII)
1^a^	Abnormal facial sensation perception (V)
1^a^	Abnormal chewing and muscle tone (V)
1^a^	Ear droop, eyelid droop, upper lip droop
	Absence of palpebral reflex and menace response (VII)
1^a^	Abnormal swallowing (IX, X)
1^a^	Abnormal tongue size and asymmetry (XII)

^a^one point for each side (right/left)

### Sample collection and analysis

All procedures were conducted in sterile conditions to exclude sample contamination. With the exception of blood samples, all biological specimens were collected with the animal sedated with xylazine (Rompun®, Bayer Health Care, Monheim, Germany) (IV 0.05 mg/kg). Two blood venous samples (5 mL/aliquot) were collected from the jugular vein of each animal: one aliquot was collected in an EDTA tube for hemocromocytometric analysis, the other was collected into a serum vacuum tube for biochemical profile (alanine transaminase [ALT], aspartate transaminase [AST], alkaline phosphatase [ALP], creatine phosphokinase [CPK], gamma-glutamyltranferase [GGT], total bilirubin, blood urea nitrogen [BUN], creatinine, glucose, total serum protein, albumin, total calcium, magnesium, sodium, phosphorus, and potassium concentrations).

Cerebrospinal fluid (CSF) was aseptically collected from the lumbosacral site of each animal, as previously described [19]. CSF analysis was performed within 1 h of collection. Total nucleated cell count, differential cell count and total protein concentration were assessed.

Nasal and aural swabs were collected using sterile swabs from both nares and ears of each animal. Tracheobronchial aspiration (TBA) was performed as previously described [20]. Ear exudate was collected by means of a sterile disposable dog catheter (2.0 × 500 mm. Buster®; Kruuse, Langeskov, Denmark) gently inserted through a sterile otoscope speculum into the affected ear canal and flushed with 2.5 mL of warm sterile saline solution. The aspirated fluid was then placed in a sterile tube. Aural, nasal and tracheobronchial samples were stored at 0–4 °C until processing.

Bacterial cultures and *M. bovis* identification were carried out at the Istituto Zooprofilattico Sperimentale della Lombardia e dell’Emilia Romagna (Brescia, Italy). Blood agar, brain heart infusion agar and MacConkey agar were used for bacteriological cultures. Cultures were incubated with 10 % CO_2_ at 37 °C for 24/48 h. *Mycoplasma* cultures were performed on PPLO (pleuropneumonia-like organisms) bovine agar and broth and incubated for 7 days at 37 °C in microaerophilic condition. *M. bovis* was identified by polymerase chain reaction (PCR) assay according to a validated method [21].

### Treatment

All animals underwent a standardized therapeutic protocol at T0. Each calf received tulathromycin (Draxxin®; Zoetis) (single subcutaneous injection of 2.5 mg/kg), oxytetracycline hydrochloride (Terramicina 100®; Zoetis) (intramuscular injection of 9.27 mg/kg for 5 consecutive days), and carprofen (Rimadyl®; Zoetis) (subcutaneous injection of 1.4 mg/kg, repeated the fourth day). The referring veterinarian was instructed to continue the treatment from day 6 to 14 with an oral formulation containing oxytetracycline hydrochloride (10 mg/kg twice a day).

### Statistical analysis

Statistical analysis was performed using the R2 1.0 freeware statistical software package (R Commander 2.0–4). The Shapiro-Wilk normality test was used to determine normal data distribution. Numerical data are presented as mean, frequency and/or percentage. A pairwise Wilcoxon rank sum test was used to assess the effect of treatment on clinical and neurological findings in relation to the experimental time points. Statistical significance was set at *P* ≤ 0.05 and Bonferroni correction was applied for multiple comparisons.

## Results

Twenty-two calves were enrolled in the study. The animals (18 males and 4 females, mean age 77 days, range 20–180) came from eight different herds. The breed types were Italian-Holstein Friesian (*n* = 9, 41 %), Piedmontese (*n* = 3, 13 %), Limousine (*n* = 1, 5 %), Belgian Blue (*n* = 1, 5 %), and cross-breed (*n* = 8, 36 %); 16 (73 %) were referred during the autumn and winter and the remaining 6 (27 %) during the spring and summer months.

### Physical and neurological examination

**Fig. 1:**
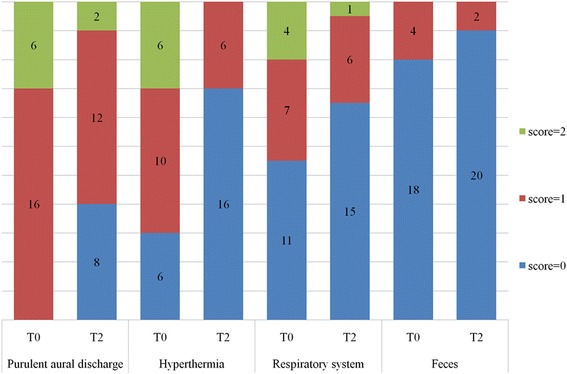
Most common clinical findings at diagnosis (T0) and at 2 weeks after therapy (T2)

**Fig. 2:**
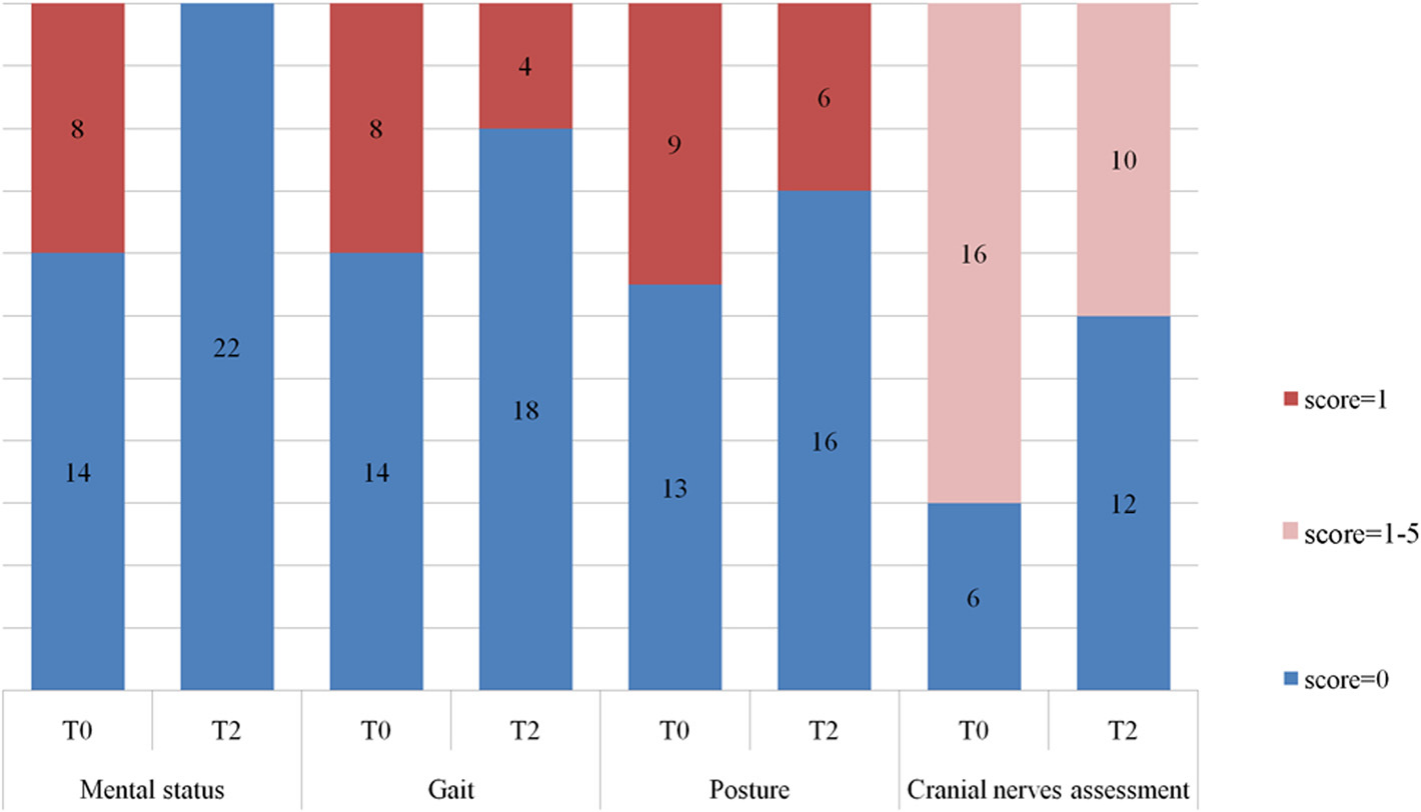
Most common neurological findings at diagnosis (T0) and 2 weeks after therapy (T2)

Physical and otoscopic examination at T0 revealed monolateral and bilateral otorrhea in 16 and 6 calves, respectively. Endoscopic examination was carried out by a single endoscopist (PG) at T0 in 11 calves. The tympanic membrane could not be visualized in any of the animals. Seventeen (77 %) animals had abnormal neurological findings and five did not. The peripheral vestibular system was involved in all calves presenting with neurological signs. None of the animals showed signs of arthritis or a cranial nerve score >5. Figures 1 and 2 present the clinical and neurological findings at T0 and T2.

**Fig. 3:**
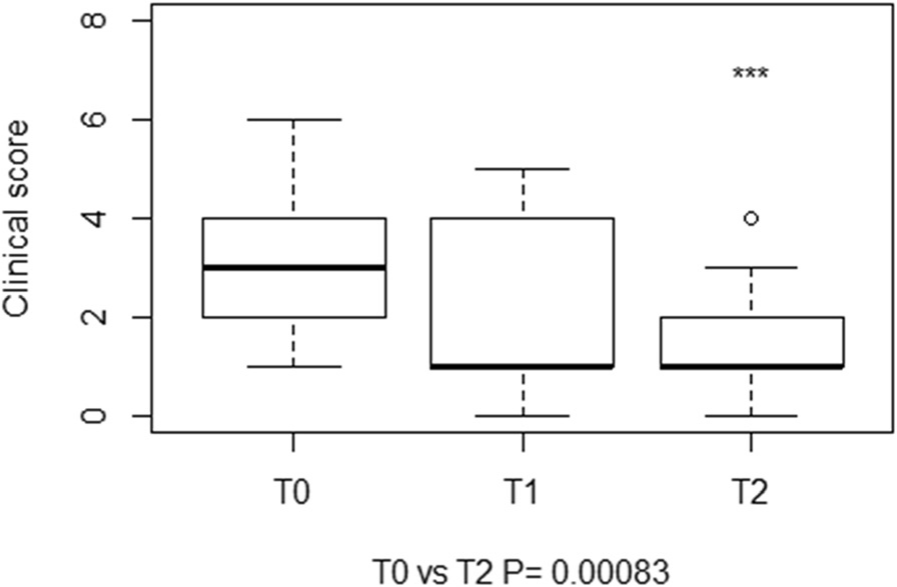
Boxplots representation of clinical scores at T0, T1 and T2. The boxplots depict the minimum and maximum values, the upper (Q3) and lower (Q1) quartiles and the median. The median is identified by a line inside the box. The length of the box represents the interquartile range. Values that deviate appreciably from most of the measurements in the data set are labeled as outliers (o)

**Fig. 4:**
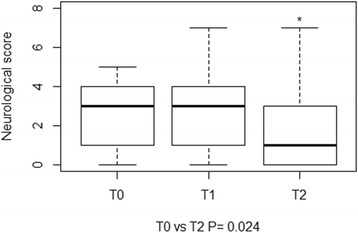
Boxplots representation of neurological scores at T0, T1 and T2. The boxplots depict the minimum and maximum values, the upper (Q3) and lower (Q1) quartiles and the median. The median is identified by a line inside the box. The length of the box represents the interquartile range. Values that deviate appreciably from most of the measurements in the data set are labeled as outliers (o)

**Fig. 5:**
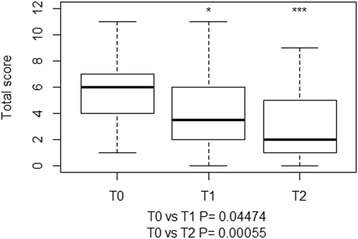
Boxplots representation of the final total scores assigned at T0, T1, and T2. The boxplots depict the minimum and maximum values, the upper (Q3) and lower (Q1) quartiles and the median. The median is identified by a line inside the box. The length of the box represents the interquartile range. Values that deviate appreciably from most of the measurements in the data set are labeled as outliers (o)

Head tilt and vestibular ataxia were the only posture and gait abnormalities observed, respectively. Positional strabismus and motor dysfunction of facial nerve (VII), alone or in combination, were the most common findings on cranial nerve assessment at each time point. A significant difference in the clinical (Fig. 3) and neurological (Fig. 4) scores was found between T0 and T2. A significant difference in the final total score was found between T0 and T1, and T0 and T2 (Fig. 5). Deterioration of neurological conditions with central vestibular system deficits occurred in 1 calf between T0 and T1. For welfare reasons, the animal was treated with a different therapeutic protocol and excluded from assessment at T2.

A significant improvement of clinical and neurological scores was observed in 20 (90 %) calves, a full recovery in only 1 (5 %). No worsening of clinical conditions was observed or further treatments required at the one-month follow-up. The agreement between neurological deficits and otoscopy was 77 %.

### Laboratory findings

Hemocromocytometric and serum biochemical findings were unremarkable. CSF analysis of samples from 21 calves was abnormal in 5 (reference limits: total cell count <10 cells/μL; microproteins <40 mg/dL). One sample was excluded due to severe blood contamination. Mild mononuclear pleocytosis was observed in CSF samples from 2 calves (total cell count 12 cells/μL and microproteins 20.53 mg/dL; total cell count 11 cells/μL and microproteins 20.94 mg/dL, respectively). Neutrophilic pleocytosis was found in CSF samples from 2 other calves (total cell count 95 cells/μL and microproteins 163.2 mg/dL; total cell count 50 cells/μL, microproteins 43.91 mg/dL, respectively). Albuminocytological dissociation was detected in 1 animal (total cell count 8 cells/μL; microproteins 72.3 mg/dL). Table 3 presents the results of bacterial and mycoplasmal cultures from affected ears, upper and lower respiratory tract.

**Table 3:** Results of bacterial and mycoplasmal cultures

Bacterial colture results	Affected Ears		Respiratory tract	
	Swabs	Lavages	Swabs	TBA
	*n* = 28	*n* = 28	*n* = 22	*n* = 20
*M. bovis*	16	18	14	9
*M. bovis* and *P. multocida*	5	5	2	3
*M. bovis* and *Streptococcus* spp.	1	-	1	-
*M. bovis* and *Staphylococcus* spp.	-	1	-	-
*M. bovis* and *P. multocida* + *Streptococcus* spp.	-	-	1	-
*M. bovis* and *Pseudomonas* spp.	-	1	-	-
*Mycoplasma* spp.	-	-	2	1
*P. multocida*	1	-	-	-
No bacteria	4	1	1	7
Contaminated samples (not diagnostic)	1	2	1	-

Aural swabs and lavages tested positive for *M. bovis* in 79 and 89 % of cases, respectively; the observed agreement between samples from aural swabs and lavages was 77 %. *M. bovis* was isolated in 3 out of 16 aural swabs collected from clinically healthy contralateral ears in association with *Streptococcus* (*n* = 1), *P. multocida* (*n* = 1), and mixed bacterial flora (*n* = 1), respectively. Table 4 reports the distribution of the causative agents isolated from the ears and respiratory tract. Overall, *M. bovis*, either alone or together with another bacterial agent, was isolated from the upper and/or lower respiratory tract in 19 (86 %) calves; *M. bovis* was isolated from the upper and/or lower respiratory tract in 10 out of 11 calves with respiratory signs.

**Table 4:** Distribution of the causative agents isolated from the ears and respiratory tract

Respiratory tract	*M. bovis*	*M. bovis* +	*M. bovis* +	*M. bovis* +	*Mycoplasma* spp.	Other mixed bacteria
		*P. multocida*	*Streptococcus* spp.	*Streptococcus* spp. *+*		
Ears						
				*P. Multocida*		
*M. bovis*	9 (4^a^; 1^b^; 4^c^)	1^b^	-	1^a^	1^c^	1^a^
*M. bovis* +	2^c^	3 (1^a^; 1^b^; 1^c^)	-	-	1^a^	-
*P. multocida*						
*M. bovis* +	1^a^	-	-	-	-	-
*Staphylococcus* spp.						
*M. bovis* +	-	-	1^a^	-	-	-
*Streptococcus* spp.						
*M. bovis* +	1^a^	-	-	-	-	-
*Pseudomonas* spp.						

^a^nasal swabs

^b^TBA

^c^nasal swabs and TBA

## Discussion

With this study we evaluated, in field conditions and by means of a simple scoring system, clinicopathological findings in calves with OM/OMI and the clinical efficacy of a standardized therapeutic protocol. Also, we identified associated causative agents and their possible correlation with concurrent BRD. As in previous studies [2, 5, 6], veal calves made up the majority of the study population, though the incidence of OMI in dairy calves seems to be rising [6, 10]. Most reports state that males and females seem to be equally susceptible to the infection [7, 8, 10]; however, Lamm et al. [7] reported that males are more prone to *M. bovis* infection because they are fed poor quality milk and do not receive adequate colostrum. In the present study, the males far outnumbered the females (18 vs. 4) which is likely to be related with the type of breeding (veal calves) since males are reared more often than females. Seeking possible correlations between the consumption of wasted milk and the pathogenesis of OMI was beyond the scope of this study. Inclement weather seems to be a predisposing factor for the development of OM/OMI [5–9]. Indeed, the majority (73 %) of the calves were referred during the autumn and winter months.

The presenting complaints/clinical signs, neurological signs, and the frequency of unilateral/bilateral ear involvement were consistent with previous observations [1, 7, 8, 10, 11, 15]; moreover, the persistence of mild clinical and neurological signs at T2 did not affect the animals’ mental status, appetite or weight gain, as ascertained by telephone interviews with the owners. These findings are in line with evidence that animals tend to adapt and thrive despite the persistence of neurological signs [8, 10, 22]. Persistent head tilt seems to be a problem only in valuable animals [8, 10].

Scoring systems for the evaluation of gait, acute postoperative pain, and sepsis in cattle have been validated and found to be reliable and sensitive for assessing the severity of clinical signs [23, 24]. To the best of our knowledge, there are no validated scoring systems for evaluating clinical signs and/or response to therapy in calves with OM/OMI. Therefore, we applied two numerical scoring systems to objectively quantify clinical and neurological findings, respectively, at each experimental time point.

Abnormal CSF analysis results are suggestive of central nervous system (CNS) involvement [8]. None of the calves in the present study showed neurological signs consistent with central vestibular disease; however, the CSF abnormalities in five animals could be explained by a focal spread of infection to the CNS. Indeed, the inner ear communicates with the subarachnoid space through the cochlear canaliculus [25]. The use of diagnostic imaging, such as computed tomography (CT) or magnetic resonance imaging (MRI), could help to clarify this [26]. Unfortunately, this was not possible since the study was conducted in the field.

To date, different therapeutic approaches have been used in calves for the treatment of OM/OMI, but prospective studies evaluating the efficacy of a standardized protocol are lacking. Furthermore, because there are no licensed drugs for the treatment of these disorders, active against Mycoplasma spp., in calves, optimal therapy remains to be determined and extra-label drug use is required [1, 8]. For the purpose of this survey, however, a randomized, double-blind placebo control study was not designed for ethical reasons and animal welfare, since meningitis and meningoencephalitis can develop secondary to the extension of infection [1, 10].

Our results suggest that, under the standardized therapeutic protocol applied in this study, the combined use of tulathromycin (Draxxin®; Pfizer), oxytetracycline hydrochloride (Terramicina 100®; Zoetis), and carprofen (Rimadyl®, Zoetis) might be effective for the treatment of OM/OMI in calves. Significant clinico-neurological improvement was observed after 2 weeks of treatment in 95 % of the calves and full recovery was achieved in only one animal. Prompt initiation of therapy (within 12–24 h after the onset of clinical signs) was probably key to symptom improvement since prognosis seems to be more favorable when therapy is begun early in the course of the disease [2, 9, 27].

Tulathromycin, a novel, semi-synthetic macrolide bacteriostatic antimicrobial, is active against *M. bovis* and other bacterial respiratory pathogens in cattle. Following parenteral administration, it is rapidly adsorbed and widely distributed, and has a long elimination half-life in lung tissue [28]. It acts by inhibiting protein synthesis in susceptible microorganisms. Once penetrated into the bacterial cell, it irreversibly binds to receptors of the 30S ribosomal subunits [29]. In a retrospective study [10], tulathromycin was administered for the treatment of OMI in calves, but no data about the number of treated animals or its efficacy are available. Oxyteracycline hydrochloride, a bacteriostatic, broad-spectrum tetracycline antibiotic, is widely distributed throughout the body. Although resistance of European mycoplasma strains to oxytetracycline has been reported [30, 31], it is effective against bacteria causing OMI, especially with prolonged use [11]. It acts similarly to tulathromycin by irreversibly binding to receptors of the 50S ribosomal units [29]. In the present study tulathromycin and oxitetracycline hydrochloride have been used together as the association between bacteriostatic antibiotics can determine the summation of their individual effects [29]. Carprofen, a nonsteroidal anti-inflammatory drug, was added to antibiotic therapy to reduce rectal temperature and pain [1]. To date, combined use of anti-inflammatory drugs and antibiotics has been evaluated only in the treatment of BRD complex [32–34]; however, its efficacy is controversial [35].

As reported elsewhere, *M. bovis*, either alone or in association with *P. multocida*, *Streptococcus* spp., *Staphylococcus* spp. and *Pseudomonas*, was the most frequently isolated pathogen [7, 8, 10]. Indeed, it has been demonstrated that under opportune conditions many bacterial species colonize the middle ear [1]. Respiratory tract infections, often with the same pathogen, have been reported in calves with OMI [1, 2, 8]. Also in this study *M. bovis* was isolated from the upper and/or lower respiratory tract in 10 out of 11 calves with respiratory signs in which the OMI may have developed secondary to respiratory disease. The most common risk factor for OMI appears to be respiratory tract infections [1]: respiratory pathogens colonize the pharynx and tonsils and eventually the auditory tube, which represents the major route of infection in OMI [1]. The isolation of *M. bovis*, either alone or in association with other bacteria, from the aural swabs taken from the clinically healthy ears (*n* = 3) is in agreement with previous reports in which *Mycoplasma* spp. was harbored in the ears of apparently healthy animals [1]. As the host-mycoplasma relationship is complicated and many aspects of these interactions are poorly understood [36], further studies are needed to understand the role of this pathogen when its presence does not result in disease.

## Conclusion

In conclusion, this is the first prospective study to evaluate the effectiveness of a standardized therapeutic protocol for the treatment of OM/OMI in calves. A significant improvement in symptoms was observed in the majority of the calves. The persistence of mild clinico-neurological signs did not compromise the animals' productive performance. The numerical scoring system for assessing clinical and neurological signs was a useful tool to objectively evaluate the response to therapy. *M. bovis* was the pathogen most often isolated. This finding should be considered when treating OM/OMI in calves. Also, respiratory tract infection should not be underrated, since it is one of the major risk factors for the development of OM/OMI.

## Abbreviations

ALP: Alkaline phosphatase
ALT: Alanine transaminase
AST: Aspartate aminotransferase
BUN: Blood urea nitrogen
BRD: Bovine respiratory disease
CBC: Complete blood count
CNS: Central nervous system
CPK: Creatine phosphokinase
CSF: Cerebrospinal fluid
CT: Computed tomography
GGT: Gamma-glutamyltranferase
MRI: Magnetic resonance imaging
OM: Otitis media
OMI: Otitis media-interna
TBA: Tracheobronchial aspiration

## Footnotes

1 Calf Respiratory Scoring Chart http://www.vetmed.wisc.edu/dms/fapm/fapmtools/8calf/calf_respiratory_scoring_chart.pdf
